# Preliminary Evidence of a Horizontal Transfer of Paramutation Phenomenon at the *pl1* Gene in Maize (*Zea mays* L.)

**DOI:** 10.3390/plants14010011

**Published:** 2024-12-24

**Authors:** Roberto Pilu, Martina Ghidoli, Alessia Follador, Alessandro Passera, Paola Casati, Ervane Laure Cheyep Dinzeu, Elena Cassani, Michela Landoni

**Affiliations:** 1 Department of Agricultural and Environmental Sciences—Production, Landscape and Agroenergy, University of Milan, Via Celoria 2, 20133 Milan, Italy; martina.ghidoli@unimi.it (M.G.); alessia.follador@unimi.it (A.F.); alessandro.passera@unimi.it (A.P.); paola.casati@unimi.it (P.C.); ervanelaure.cheyepdinzeu@studenti.unimi.it (E.L.C.D.); elena.cassani@unimi.it (E.C.); 2 Department of Earth and Environmental Sciences, University of Pavia, Via S. Epifanio 14, 27100 Pavia, Italy

**Keywords:** paramutation, silencing, horizontal transfer, anthocyanin, *pl1*, *purple plant1*, Naturally Induced Gene Silencing, NIGS

## Abstract

Paramutation, a specific epigenetic phenomenon first identified in *Zea mays* by Alexander Brink in the 1950s, has since been observed in different plant and animal species. What sets paramutation apart from other gene silencing processes is its ability for one silenced allele (referred to as paramutagenic) to silence another allele (paramutable) in trans. The resultant silenced allele (paramutated) remains stable across generations, even after separating from the paramutagenic allele, and acquires paramutagenic properties itself. This hereditary behavior facilitates the rapid dissemination of specific gene expression patterns or phenotypes within populations, disrupting the Hardy–Weinberg equilibrium even without other evolutionary pressures. Despite extensive research, the molecular mechanisms underlying paramutation are still not fully understood, although numerous studies suggest the involvement of RNA-mediated changes in DNA methylation and in the chromatin structure of silenced genes. In this paper, we report preliminary evidence regarding horizontal paramutation transfer at the *pl1* (*purple plant1*) regulatory gene involved in the accumulation of anthocyanin in several plant tissues such as tassel, kernel, and cob. A paramutated *pl1′* allele arose spontaneously in a *pl1* population, and in this study, we found evidence of silencing events spatially associated in the field, suggesting a possible horizontal transfer of silencing among nearby plants.

## 1. Introduction

The study of genetic control of the anthocyanin pathway in maize has provided a significant contribution to the advancement of basic genetic knowledge of phenomena such as hybrid vigor, genetic regulation mechanisms, the discovery of transposons, and gene silencing [1]. The regulation of the anthocyanin pathway involves two types of transcription factors controlled by the *bHLH* and *MYB* gene families. Within the *bHLH* gene family, there are *r1* (*red color1*) and *b1* (*booster1*) genes, while the *MYB* gene family includes *c1* (*colored aleurone1*), *pl1* (*purple plant1*), and *p1* (*pericarp color1*) [2,3]. All of these may undergo spontaneously a phenomenon of irreversible gene silencing called paramutation [4,5]. Paramutation is an epigenetic phenomenon in which the epigenetic state of one allele (referred to as paramutagenic) is transferred to another allele (paramutable) in trans, resulting in a heritable modification of its gene expression at a frequency of up to 100%. The paramutable allele then acquires the ability to induce paramutation in future generations. Unlike typical mutations, where changes in the DNA sequence often lead to gene inactivation, paramutation produces various epialleles with silenced gene activity, resulting in diverse phenotypes [4,5]. Furthermore, unlike a mere phenomenon of inheritable gene silencing, the phenomenon of paramutation grants the silenced allele the ability to “pass on the silencing” to the other allele in trans, thus modifying the second Mendel’s law of classical genetics and the principle of the Hardy–Weinberg equilibrium of allele frequencies in populations [6]. This phenomenon, discovered in the last century [7,8], is poorly understood from a molecular perspective to this day, and experimental evidence suggests that different mechanisms are involved in plants and metazoans, likely due to differences in reproductive biology [9]. The mechanism through which paramutated genes are silenced across generations remains elusive; however, research across various maize systems, such as the *b1* [10,11], *p1* [12], and *pl1* [13] genes, suggests that an RNA-dependent RNA polymerase (mop1, mediator of paramutation1) is required for paramutation in maize and that specific types of small RNAs, including those defining cytosine methylation patterns, may play significant roles in this process. Hence, it is hypothesized that specific types of small RNAs are involved, capable of triggering heritable transcriptional gene silencing. In recent years, extracellular RNA (exRNA), has been attracting researchers’ attention as emerging research indicates that these RNAs are predominantly found either freely in the extracellular environment or bound to RNA-binding proteins, with only a small fraction detected within extracellular vesicles (EVs). Despite their scarcity, small RNAs associated with EVs have drawn attention in exRNA research for their potential role in facilitating trans-kingdom RNA interference (RNAi). Consequently, non-vesicular exRNAs have only recently gained attention [14]. Horizontal transfer of genetic information has become a promising useful tool for practical purposes, such as protecting plants from pathogen attacks without altering the plants’ genome and, hence, subjecting them to the stringent regulations applicable to transgenic plants [15]. Several studies have reported the possibility, through Spray-Induced Gene Silencing (SIGS), of inducing changes in the expression pattern of genes involved in resistance to viruses [16], fungi [17], and insects [18] by administering RNA molecules. In this study, we report for the first time, to our knowledge, the horizontal transfer of gene silencing at the *pl1* gene related to the phenomenon of paramutation. The results obtained lead us to hypothesize that physical contact or proximity between plants may likely convey RNA molecules capable of spreading the signal of inheritable transcriptional silencing throughout space. The identity of the vector capable of conveying the silencing signal remains unknown but it can be hypothesized that the involvement of roots apparatus during the early stages of plant development may be necessary to explain the observed phenomenon.

## 2. Materials and Methods

### 2.1. Plant Materials

In this work, we used a three-way hybrid obtained by crossing *B1 Pl1 br2* homozygous line (used as female plant) with B73/Mo17 hybrid.

The sources of the dominant anthocyanin biosynthesis regulators were the genes *booster1 (b1)* and *purple plant1 (pl1),* which determine anthocyanin accumulation in different plant tissues such as tassel, pericarp, kernel, roots, etc., [3,19,20,21] and are in the homozygous state in the female parent of the three-way hybrid. We selected this genetic background due to the high frequency of paramutation phenomena, ranging from 10^−2^ to 10^−3^ (unpublished result of our laboratory). In this genotype, the *brachytic 2* recessive mutation was also present, conferring short stature [22]. The breeding activities and the experimentation were conducted at the experimental farm “Angelo Menozzi” located at Landriano (PV), Italy (45°18′ N; 9°15′ E), 88 m a.s.l.

The field was established by sowing approximately 1500 seeds (6 rows of 50 m × 0.70 m; distance between seeds: 0.2 m).

### 2.2. Detection of Paramutation Events

Hybrid plants with colorless anthers were identified at maturity, and the distance from the beginning of each row (x/y coordinates) was recorded. The plants were tagged and at the end of the season, and each ear was harvested, dried, and scored for paramutation events (ears and seeds colorless). We used the *br2* recessive mutation to individuate and discard brachytic off-types due to unwanted self-pollination of female plants *B1 Pl1 br2* during artificial pollination. All the ears were shelled and seeds were counted by Automatic Seed Counter (TOP Cloud-agri, Hangzhou, China).

To confirm the paramutation events, 100 seeds were collected from 115 colorless or weakly pigmented ears and from 10 non-paramutated red ears as controls. The seeds were germinated in a growth chamber, in the dark, in Plexiglas boxes, on Whatman filter paper soaked with water, at 26 °C. After five days, the plantlets were exposed to light with a 16/8 photoperiod at 24 °C and after an additional 5 days, the plantlets were scored.

Four cool white fluorescent lamps (F36T12/CW/TO from GTE SYLVANIA, Lighting Products Group, Danvers, MA, USA) were used as light sources. The light intensity was 0.785 μE m^2^ s^−1^ nm^−1^.

### 2.3. Spectrophotometric Determination of Anthocyanin Content

Ten seeds of non-paramutated and paramutated plant progenies (from 3 different ears) were germinated on filter paper, as reported in Section 2.2. After five days of growth in the light, the more pigmented roots were sampled and stored at −20 °C until pigment extraction.

The anthocyanin extraction was performed on weighed roots that were homogenized with quartz sand and 2 mL of extraction buffer (1% HCl, 95% ethanol). The samples were then centrifuged at 13,000 rpm for 10 min, and 200 μL of the supernatant was collected. Their absorbance was determined spectrophotometrically at 530 nm. The amounts of anthocyanins were calculated as cyanidin 3-glucoside equivalents (ε 26,900 Lm^−1^ mol^−1^, M. W. 442.2). The analyses were conducted on three replicates for each genotype.

### 2.4. Gene Expression Analysis

Seeds collected from three red (non-paramutated) and three colorless ears (paramutated) were germinated on filter paper and root samples were obtained from two seedlings for each ear: a secondary root of around 3 cm in length was collected from each of these plants and the RNA was extracted using the Nucleozol reagent (Takara Bio, Mountain View, CA, USA). The RNA samples were then treated with DNAse (New England Biolabs, Ipswich, MA, USA) and retrotranscribed using the MultiScribe reverse transcriptase (Thermo Fisher Scientific, Waltham, MA, USA) and random hexamer primers.

The *pl1* gene expression was analyzed through real-time PCR. The amplification of *pl1 transcript* was carried out using the PL6/PL8 primer pair [19] with the following mix: PowerSYBR Master Mix 1x (Applied Biosystems, Foster, CA, USA), PL6 primer 200 nmol, PL8 primer 200 nmol, 2 µL of cDNA, and water to reach a volume of 20 µL. The *18S* gene, used as standard, was amplified using the Prun18S-F/Prun18S-R primer pair and the Prun18S-Taq probe [23] with the following mix: TaqMan Master Mix 1x (Applied Biosystems, Foster, California, USA), Prun18S-F primer 900 nM, Prun18S-R primer 900 nM, Prun18S-Taq probe 200 nM, 2 µL of cDNA, and water to reach a volume of 20 µL. The thermal cycle program for both reactions included 10 min of initial denaturation at 96 °C followed by 40 cycles of 95 °C for 15 s and 65 °C for 30 s. The amplification reactions were carried out in a StepOnePlus Real-Time PCR thermocycler (Thermo Fisher Scientific, Waltham, MA, USA). Each sample was amplified in technical triplicates for both *pl1* and *18S* genes.

Relative expression of *pl1* gene was then calculated through the 2^−ΔΔCt^ method [24] and expressed as fold-change, calculated as Log_2_(2^−ΔΔCt^).

### 2.5. Data Analysis

Microsoft Excel^®^ was used to collect the data. The PAST program (Paleontological Statistics, version 4.12) and IBM SPSS version 20 software were used to perform the statistical analysis. We used Kernel Density Estimation (KDE) statistical test to calculate probability density; this is a non-parametric approach to approximating the probability density function of a random variable, employing kernels as weighting factors. We used the “Nearest neighbors analysis” with wrap-around edge correction and the “point events” to perform statistical analysis, respectively, on 2D (X/Y coordinates) and 1D variables (Y coordinate).

For the gene expression analysis, results were compared through a one-way ANOVA, followed by Tukey’s post hoc test (*p* < 0.05).

## 3. Results

With the aim of verifying the possibility of horizontal transfer (from plant to plant) of the paramutation phenomenon at the *pl1* locus, we set up an experimental field with approximately 1500 plants. The plants used for the experimentation were heterozygous at the *pl1 locus* (see the Materials and Methods Section). We used a line that undergoes high spontaneous onset of germline or para-germline silencing, meaning that the entire plant will be silenced at the *pl1* locus.

**Figure 1:**
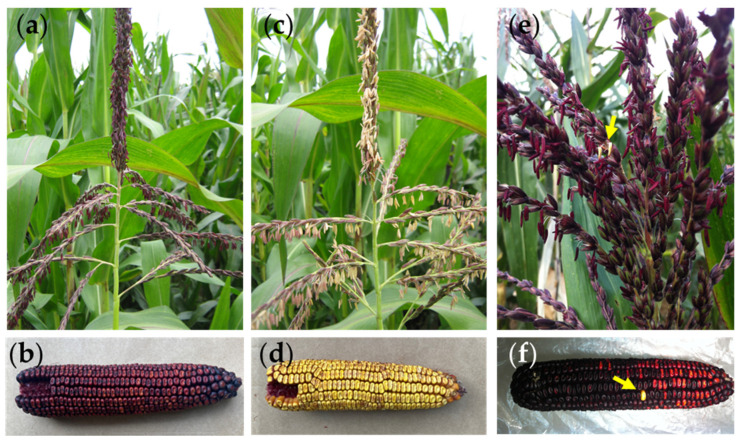
Paramutation phenomena at *pl1 Zea mays* locus. Anthocyanin accumulation in the tassel anthers (**a**) and pericarp kernel (**b**) driven by the presence of dominant *Pl1* allele. Germinal or para-germinal paramutation/silencing phenomena at *pl1* gene, scored as absent or weak pigmentation of the tassel (**c**) and pericarp kernel (**d**). Yellow arrows indicated somatic paramutation/silencing events occurring at a late developmental stage in the anthers tassel (**e**) and pericarp kernel (**f**).

**Figure 2:**
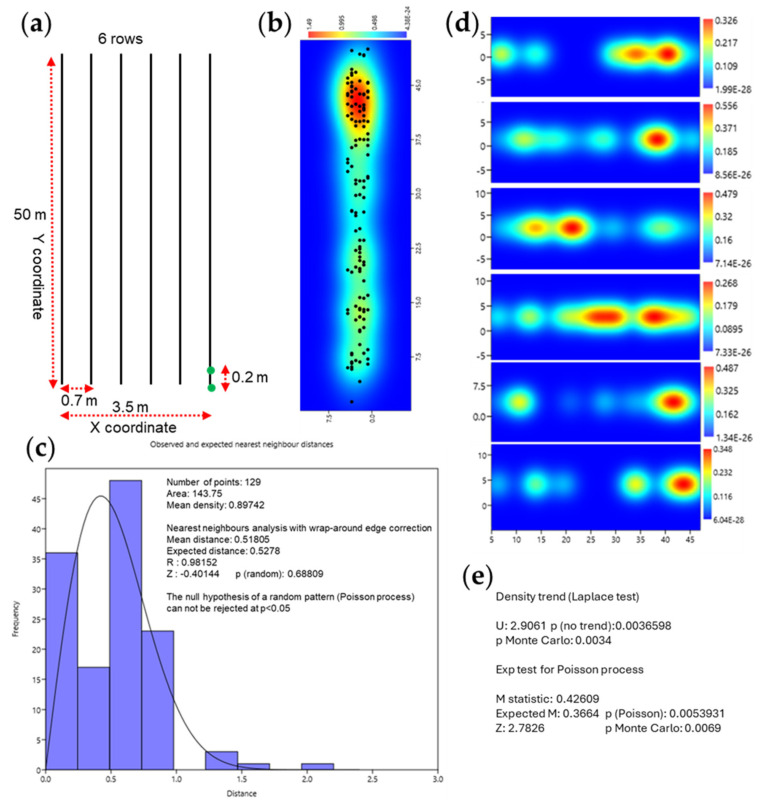
Statistical analysis regarding the appearance of silencing/paramutation at *pl1* gene scored as colorless tassels. Scheme of the field used in the experimentation; green dots indicate the distance of the plants on the rows (**a**). Probability density chart calculated by “Kernel Density Estimation” using X/Y coordinates of silencing/paramutation plants present in all the field; dots indicate the single event of silencing/paramutation (**b**). Statistical analysis “Nearest neighbors analysis” with wrap-around edge correction regarding the probability of silencing/paramutation phenomena considering all the 6 rows present in the field (**c**). Probability density chart calculated by “Kernel Density Estimation” using Y coordinate of silencing/paramutation plants present on single row (**d**). Statistical analysis performed on single row by “point events” (Y coordinate) (**e**).

At maturity, we scored the anther color (colorless vs. red). From a genetic standpoint, the hybrid should be uniformly colored due to the presence of the dominant *Pl1* allele in heterozygosity (Figure 1a,b). Hence, each plant present in this population that carried colorless anthers represented a spontaneous germinal or para-germinal paramutation/silencing phenomena at the *pl1* locus and should be distributed randomly in the field (Figure 1c,d). We also observed somatic paramutation/silencing events that occurred at a late developmental stage in the anthers, tassel, and seed (Figure 1e,f). We found 129 plants with yellow tassels (all anthers were yellow) out of 1453, and we recorded the X/Y coordinates of each plant present in the field (Figure 2a). To test the hypothesis that silencing events were randomly distributed in the field, we conducted two types of statistical analyses for events distributed across the entire field (X/Y coordinates) and for events distributed along the row (Y coordinate).

The first analysis reported in Figure 2b shows the probability density chart (using the X/Y coordinates) of silencing/paramutation plants present in the field; however, despite appearances, the “Nearest neighbors analysis” statistical test, shown in Figure 2c, did not reject the null hypothesis of a random distribution. However, in the second test, which considered the rows as a sequence of 300 linear meters of plants (6 rows × 50 m = 300 linear meters) by using the “point events test” (Y coordinate), we can reject the null hypothesis of a random distribution of the silenced plants (Figure 2d,e). In order to confirm that all plants with colorless or weakly pigmented inflorescences were events of silencing/paramutation, at the end of the season, the ears of all plants in the field were collected. All ears collected from plants with colorless tassels were found to be colorless or weakly pigmented, while all others were found to be pigmented, confirming that the origin of the *pl1* silencing was germline or para-germline. Furthermore, 100 seeds derived from 115 unpigmented tassel plants were germinated, and in all these offspring seedlings, the absence of pigmentation in the primary roots was observed, confirming that the silencing was inheritable, as expected for this phenomenon.

Moreover, to verify the silencing at the *pl1* locus, an RT-PCR analysis was conducted on root tissue collected from a subset of 10 silenced offspring seedlings. The results obtained confirmed that indeed, on the basis of the observed phenomenon, there was a transcriptional silencing at the *pl1* locus, as expected in a paramutation phenomenon [13].

Specifically, in the case of the *pl1* gene, five genes have been identified to uphold the repression of a paramutable *pl1* allele, implicated in 24-nucleotide RNA biogenesis. Consequently, it is postulated that particular types of small RNAs are implicated, capable of initiating heritable transcriptional gene silencing. We can hypothesize that the signal passing between plants consists of short RNA molecules capable of inducing heritable transcriptional changes at the *pl1* locus.

**Figure 3:**
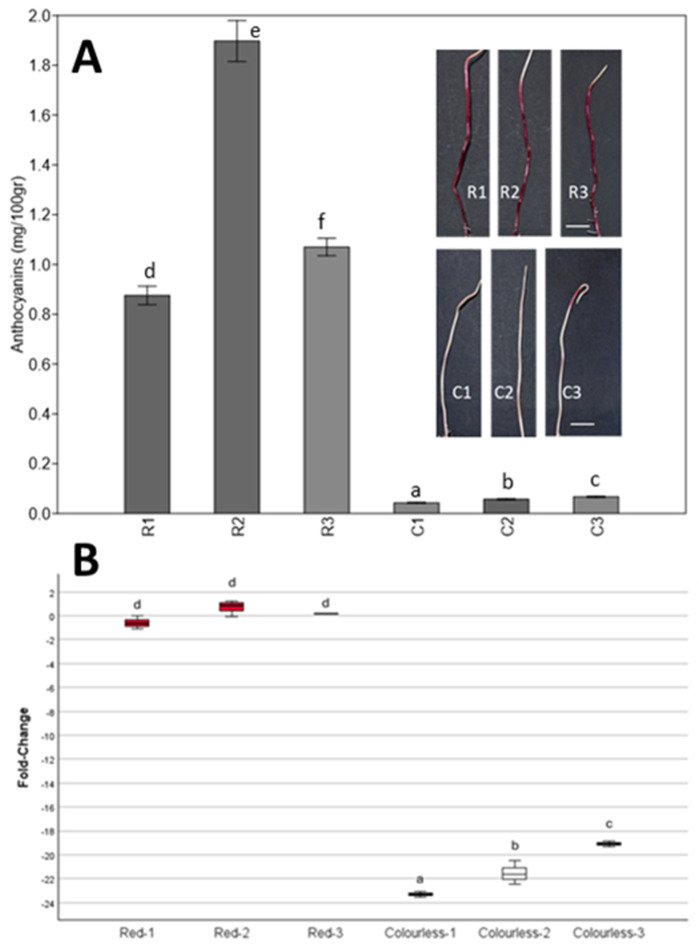
Anthocyanin quantification and expression level of the *pl1 Zea mays* gene in seedling roots. In (**A**), the root anthocyanin content is shown for three different non-paramutated plants (R1–3) compared to paramutated plants (C1–3). Bars rappresent 1cm. (**B**) Graph reporting the relative expression of the *pl1* gene in three red (Red-1, Red-2, and Red-3) and three colorless (Colorless-1, Colorless-2, Colorless-3) plants. The *Y*-axis reports the relative levels of expression as fold-change: Log_2_(2^−ΔΔCt^). Different letters above the boxes identify statistically significant differences in the results according to a one-way ANOVA followed by Tukey’s post hoc test (*p* = 0.001).

To confirm that the phenomenon is indeed caused by the silencing of the *pl1* gene, the expression level of this gene has been quantified in three separate events of colorless paramutated plants and compared to the expression level in the red plants. The expression levels are reported as fold-change in Figure 3.

It can be seen that all three considered red plants have similar expression levels of the *pl1* gene, while the three colorless plants have significantly lower levels of expression for the gene, reporting on average a −21 fold-change.

It is interesting to note that, while the red plants have no statistically significant difference between their levels of expression, each colorless plant differs from the other two, confirming that each colorless plant is a different paramutation event and that different events can vary in efficacy in silencing the gene.

## 4. Discussion

In this paper, we reported, for the first time to our knowledge, data regarding a possible horizontal transfer of paramutation phenomenon at the *pl1* gene in maize. The data collected indicate that the presence of germinal or para-germinal events of paramutation events are associated and not random as expected (Figure 2). In addition, we can hypothesize that there is a need for physical contact or proximity between the plants since statistically significant results were found in the intra-row analysis, with a distance of 0.2 m between plants (Figure 2d,e), rather than considering the field surface with an inter-row spacing of 0.7 m and 0.2 m among plants (Figure 2b,c). Since all tissues of the adult plant, including the germline, are involved in the phenomenon (Figure 1c,d)—unlike what is seen in the higher frequency spontaneous silencing that leads to somatic sectors (Figure 1e,f)—we suggest that the signal for the *pl1* gene silencing is transmitted at the root level, or at least in the early stages of development. To confirm this result, an analysis of *pl1* gene expression was conducted on the progenies of silenced/paramutated plants. The results obtained, shown in Figure 3, clearly demonstrated that the *pl1* gene is expressed at lower levels in the paramutated plants, as expected in the case of *pl1* silencing at the root level (Figure 3).

The non-random distribution of silencing events led us to hypothesize that there may be a physical exchange of genetic information in the early stages of seedling development: the spontaneous event of germline silencing in a plant increases the probability that another nearby plant will undergo a para-germline silencing event. We can hypothesize that the signal passing between plants could be short RNA molecules capable of inducing inheritable transcriptional changes at the *pl1* locus.

This hypothesis is supported by recent advancements in the use of RNA molecules to modify gene expression in plants for purposes such as protection against pathogen attacks. Various studies have indicated the potential for inducing alterations in the expression profiles of genes associated with virus, fungal, and insect resistance through Spray-Induced Gene Silencing (SIGS) using RNA molecules [16,17,18]. Furthermore, we can hypothesize that physical contact, particularly root contact/anastomosis, may convey silencing signals. Of course, we cannot exclude the mediation of other vectors such as viruses, bacteria, fungi, or animals. It is well known, for example, that plant small RNAs (sRNAs) might travel through insects and nematodes [25,26]. Studies utilizing next-generation sequencing have identified plant-derived microRNAs (miRNAs) in insects [27,28]. This implies that these miRNAs, once ingested by insects, could traverse the gut and modulate gene expression in these pests and/or spread in other plants through piercing mouth-parts. Double-stranded RNAs (dsRNAs), or hairpin RNAs, seem to be the primary carriers of sRNAs for this inter-kingdom RNA interference (RNAi) phenomenon [22]. These non-coding sRNAs possess the ability to migrate between cells via plasmodesmata (PD), following a symplasmic route, and also from donor to recipient tissues through the phloem, inducing gene silencing in their designated cells. These mobile sRNAs operate within non-cell-autonomous communication networks, modulating diverse biological phenomena like plant growth, reproduction, and defense mechanisms [29].

In conclusion, for the first time, a phenomenon of horizontal transfer of paramutation regarding the *pl1* gene in maize is reported. Unlike the promising methodology regarding Spray-Induced Gene Silencing (SIGS), the observed phenomenon concerns a horizontal transfer of inheritable transcriptional silencing, which could be named Naturally Induced Gene Silencing (NIGS). Further work will be necessary to confirm the results obtained in this study and to identify the agent capable of transferring the paramutation signal between plants.

## Data Availability

Data are contained within the article.

## References

[1] Freeling M., Walbot V. (1994). The Maize Handbook.

[2] Chandler V.L., Radicella J.P., Robbins T.P., Chen J., Turks D. (1989). Two regulatory genes of the maize anthocyanin pathway are homologous: Isolation of B utilizing R genomic sequences. Plant Cell.

[3] Dooner H.K., Robbins T.P., Jorgensen R.A. (1991). Genetic and developmental control of anthocyanin biosynthesis. Annu. Rev. Genet..

[4] Pilu R. (2011). Paramutation: Just a curiosity or fine tuning of gene expression in the next generation?. Curr. Genom..

[5] Hollick J.B. (2017). Paramutation and related phenomena in diverse species. Nat. Rev. Genet..

[6] Springer N.M., MacGinnis K. (2015). Paramutation in evolution, population genetics and breeding. Semin. Cell Dev. Biol..

[7] Brink R.A. (1956). Change associated with the R locus in maize is directed and potentially reversible. Genetics.

[8] Patterson G.I., Chandler V.L., Meyer P. (1995). Paramutation in Maize and Related Allelic Interactions. Gene Silencing in Higher Plants and Re-lated Phenomena in Other Eukaryotes. Current Topics in Microbiology and Immunology.

[9] Gabriel J.M., Hollick J.B. (2015). Paramutation in maize and related behaviors in metazoans. Semin Cell Dev. Biol..

[10] Chandler V.L. (2007). Paramutation: From maize to mice. Cell.

[11] Aubert J., Bellegarde F., Oltehua-Lopez O., Leblanc O., Arteaga-Vazquez M.A., Martienssen R.A., Grimanelli D. (2022). AGO104 is a RdDM effector of paramutation at the maize b1 locus. PLoS ONE.

[12] Sidorenko L.V., Chandler V.L., Wang X., Peterson T. (2024). Transcribed enhancer sequences are required for maize p1 paramutation. Genetics.

[13] Deans N.C., Giacopelli B.J., Hollick J.B. (2020). Locus-specific paramutation in Zea mays is maintained by a PICKLE-like chromodomain helicase DNA-binding 3 protein controlling development and male gametophyte function. PLoS Genet.

[14] Borniego M.L., Innes R.W. (2023). Extracellular RNA: Mechanisms of secretion and potential functions. J. Exp. Bot..

[15] Akbar S., Wei Y., Zhang M.Q. (2022). RNA Interference: Promising approach to combat plant viruses. Int. J. Mol. Sci..

[16] Das P.R., Sherif S.M. (2020). Application of exogenous dsRNAs-induced RNAi in agriculture: Challenges and triumphs. Front. Plant Sci..

[17] Dalakouras A., Wassenegger M., Dadami E., Ganopoulos I., Pappas M.L., Papadopoulou K. (2020). Genetically modified organism-free RNA interference: Exogenous application of RNA molecules in plants. Plant Physiol..

[18] Cagliari D., Dias N.P., Galdeano D.M., dos Santos E.Á., Smagghe G., Zotti M.J. (2019). Management of pest insects and plant diseases by non-transformative RNAi. Front. Plant Sci..

[19] Pilu R., Piazza P., Petroni K., Ronchi A., Martin C., Tonelli C. (2003). plbol3, a complex allele of the anthocyanin regulatory pl1 locus that arose in a naturally occurring maize population. Plant J..

[20] Petroni K., Pilu R., Tonelli C. (2014). Anthocyanins in corn: A wealth of genes for human health. Planta.

[21] Pilu R. (2015). Paramutation phenomena in plants. Semin. Cell Dev. Biol..

[22] Pilu R., Cassani E., Villa D., Curiale S., Panzeri D., Badone F.C., Landoni M. (2007). Isolation and characterization of a new mutant allele of brachytic 2 maize gene. Mol. Plant Breed..

[23] Jawhari M., Abrahamian P., Sater A.A., Sobh H., Tawidian P., Abou-Yawdah Y. (2015). Specific PCR and real-time PCR assays for the detection and quantitation of ‘*Candidatus* Phytoplasma phoenicium’. Mol. Cell. Probes.

[24] Livak K.J., Schmittgen T.D. (2001). Analysis of relative gene expression data using real-time quantitative PCR and the 2^−ΔΔCt^ method. Methods.

[25] Mongelli V., Saleh M.C. (2016). Bugs are not to be silenced: Small RNA pathways and antiviral responses in insects. Annu. Rev. Virol..

[26] Zhu K., Liu M., Fu Z., Zhou Z., Kong Y., Liang H., Lin Z., Luo J., Zheng H., Wan P. (2017). Plant microRNAs in larval food regulate honeybee caste development. PLoS Genet..

[27] Zhang L.L., Jing X.D., Chen W., Wang Y., Lin J.H., Zheng L., Dong Y.H., Zhou L., Li F.F., Yang F.Y. (2019). Host plant-derived miRNAs potentially modulate the development of a cosmopolitan insect Pest, Plutella xylostella. Biomol. Ther..

[28] Bally J., Fishilevich E., Doran R.L., Lee K., de Campos S.B., German M.A., Narva K.E., Waterhouse P.M. (2020). Plin-amiR, a pre-microRNA-based technology for controlling herbivorous insect pests. Plant Biotechnol. J..

[29] Yan Y., Ham B.K. (2022). The Mobile Small RNAs: Important Messengers for Long-Distance Communication in Plants. Front. Plant Sci..

